# N^6^ ‐methyladenosine RNA demethylase FTO regulates extracellular matrix‐related genes and promotes pancreatic cancer cell migration and invasion

**DOI:** 10.1002/cam4.5054

**Published:** 2022-07-25

**Authors:** Wei Wang, Ying He, Lun Wu, Lu‐Lu Zhai, Long‐Jiang Chen, Li‐Chao Yao, Kai‐Huan Yu, Zhi‐Gang Tang

**Affiliations:** ^1^ Department of Hepatobiliary Surgery in East Hospital Renmin Hospital of Wuhan University Wuhan China; ^2^ The State Key Laboratory Breeding Base of Basic Science of Stomatology (Hubei‐MOST) & Key Laboratory of Oral Biomedicine Ministry of Education, School & Hospital of Stomatology Wuhan University Wuhan China; ^3^ Department of Hepatobiliary Surgery, Dongfeng Hospital Hubei University of Medicine Shiyan China; ^4^ Department of Pancreatic Surgery Renmin Hospital of Wuhan University Wuhan China

**Keywords:** extracellular matrix, m^6^A, pancreatic cancer, RNA methylation

## Abstract

Pancreatic cancer (PC) is a deadly disease, and its post‐transcriptional gene regulation mechanism remains unclear. The abundant extracellular matrix (ECM) in PC plays an important role in tumor progression. This study is the first to focus on the role of N^6^‐methyladenosine (m^6^A) RNA methylation, an emerging post‐transcriptional regulatory mechanism, in the regulation of the ECM in PC. Here, we found that ADAMTS2, COL12A1, and THBS2 were associated with the prognosis of PC by comprehensive analysis of differentially expressed genes from two independent GEO expression profile datasets and m^6^A‐related genes in RMVar database (PAAD). GO and KEGG enrichment analysis found that these m^6^A‐related targets are chiefly functionally concentrated in the ECM region and participate in ECM signal transduction. Correlation analysis revealed that these genes can be regulated by the demethylase FTO. Cell biology function assays showed that knockdown of FTO‐inhibited PC cell abilities to migrate and invade in vitro. qRT‐PCR and MeRIP experiments showed that after knockdown of FTO, the mRNA levels of ADAMTS2, COL12A1, and THBS2 and their m^6^A modification levels were significantly reduced. These results indicate that m^6^A RNA demethylation is associated with the regulation of ECM in PC. In conclusion, m^6^A RNA demethylase FTO regulates ECM‐related genes and promotes PC cell abilities to migrate and invade, our work provides a new perspective on the molecular mechanism of PC progression.

## Graphical abstract

We identified, for the first time, the important role of m6A modification in the regulation of ECM related molecules in pancreatic cancer (PC). Our data revealed that ADAMTS2, COL12A1, and THBS2 may be involved in PC metastasis and progression and that this role is regulated by the m6A demethylation transferase FTO. These molecules hold promise as novel biomarkers for clinical prognosis in PC and may be served as potential therapeutic targets of PC, and this study provides a new perspective for the development of therapeutic strategies for PC.

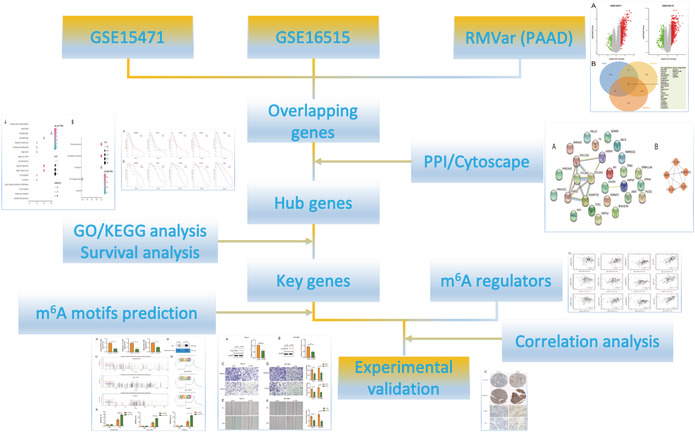

## 1 INTRODUCTION

Pancreatic cancer (PC) is one of the most malignant tumors of the digestive system, the 5‐year survival rate for PC is only 9% in the United States.^1^ Globally, hundreds of thousands of patients die of PC each year, and its mortality rate ranks fourth among malignant tumors.^2^ Among all newly diagnosed patients, only 20% of patients can benefit from a potentially curable surgical resection, and the remaining 80% suffer from unresectable locally advanced or metastatic disease.^3^ Surgery is the hope of curing PC. However, most patients with PC are already in the advanced stage of the tumor when they are diagnosed. Only <20% of patients have the opportunity of surgical excision, and 90% of them have recurrence or metastasis after surgery.^4^ Local invasion or distant metastasis is an important biological feature of PC and the main obstacle that limits radical surgical resection.^5^ Hence, it is urgent to find specific molecular targets and mechanisms that regulate invasion and metastasis of PC. Although whole‐gene expression patterns in PC have been extensively studied, the post‐transcriptional regulation of gene expression in invasion and metastasis of PC has not been fully studied. Research on post‐transcriptional gene regulation can better understand the molecular mechanism of PC and provide a more comprehensive biological understanding of PC progression.

**FIGURE 1:**
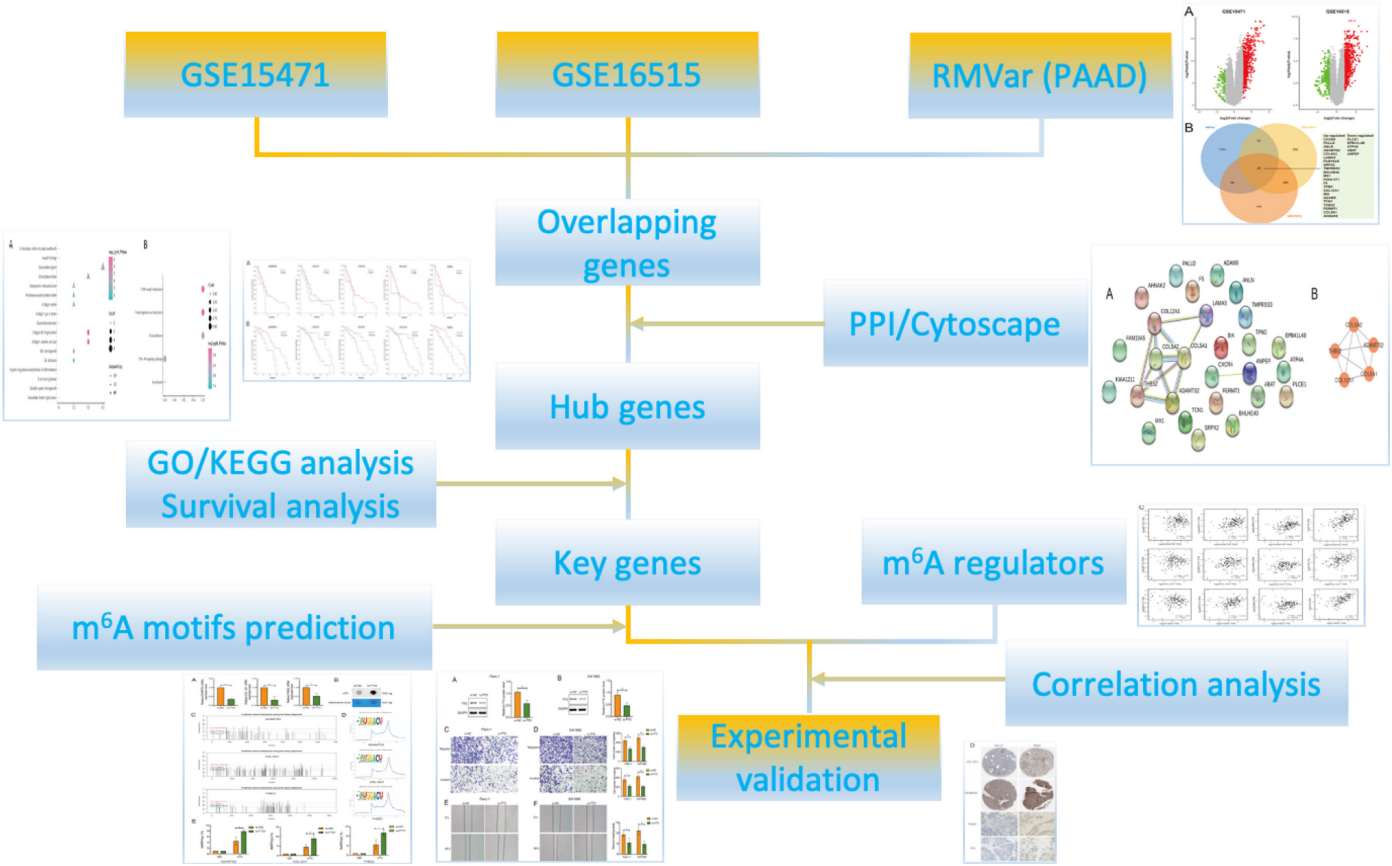
The flow chart of the present study.

N^6^‐methyladenosine (m^6^A) RNA methylation is one of the most common internal modifications in mammalian mRNA.^6^, ^7^ m^6^A methylation is dynamically reversible,^8^ and it is involved in nearly all steps of RNA metabolism such as mRNA translation, degradation, and output.^9^, ^10^ m^6^A methylation affects basic biological processes of cells, including DNA damage repair,^11^ meiosis,^12^ innate immune response,^13^ and tumorigenesis.^14^ The role of m^6^A in gastric cancer,^15^ liver cancer,^16^ and colon cancer^17^ has been reported, and m^6^A has recently been implicated in the progression of PC.^18^ However, these studies only examined the relationship between one m^6^A regulatory factor and one target gene. Too much attention to the function of a single gene may miss important changes in biological traits, and these changes are often determined by a set of functional genes.^19^ In this study, two independent GEO expression profile datasets and m^6^A‐related genes in the RMVar database (PAAD) were employed to comprehensively analyze and identify three m^6^A‐related genes, THBS2, COL12A1, and ADAMTS2, which were correlated to the prognosis of PC. Moreover, these genes can be regulated by the m^6^A RNA demethylase FTO. Gene Ontology (GO) annotation and Kyoto Encyclopedia of Genes and Genomes (KEGG) pathway analysis showed that the genes were mainly functionally concentrated in the extracellular matrix (ECM) and involved in ECM‐receptor interaction. Subsequent in vitro assays showed that inhibition of FTO expression repressed PC cell migration and invasion. Mechanistically, FTO regulates the expression of ADAMTS2, COL12A1, and THBS2 through m^6^A RNA methylation. Our study is the first to focus on the role of m^6^A modification in ECM of PC, providing new insights into the mechanism of PC metastasis. The flow chart of this study was shown in Figure 1.

## 2 MATERIALS AND METHODS

### 2.1 Microarray data and identification of m^6^A‐related genes in PC

GSE15471^20^ and GSE16515^21^ microarray data sets were obtained from Gene Expression Omnibus (GEO) database. Using GEO2R (http://www.ncbi.nlm.nih.gov/geo/geo2r/)^22^ statistical tools to calculate and evaluate the differentially expressed genes (DEGs) between PC and tissue adjacent to carcinoma, fold‐change (FC) of gene expression was calculated, with log2FC ≥ 1.5, *p* < 0.01 as the threshold value standard. The DEGs were combined with 1393 m^6^A‐related genes of pancreatic adenocarcinoma (PAAD) (after deleting the duplicate genes) downloaded from the RMVar database (http://rmvar.renlab.org)^23^ to obtain overlapping genes.

### 2.2 Protein–protein interaction (PPI) network construction and analysis

The STRING database (verson 11.5; http://string‐db.org) was employed to predict the PPI network. The result of the STRING database analysis was imported into Cytoscape to map and analyze the interactive network. MCODE (version 1.4.2)^24^ was used to identify the most important modules in the PPI network.

### 2.3 GO and KEGG analysis of the most significant module genes

The Database for Annotation, Visualization, and Integrated Discovery (DAVID, version 6.8; http://david.ncifcrf.gov) was utilized to determine the GO annotation and KEGG pathway analyses of the most significant module genes.^25^ *p* < 0.05 was considered statistically significant.

### 2.4 Key genes selection and analysis

Gene Expression Profiling Interactive Analysis (GEPIA) was used to analyze the correlation between the most significant modular genes and the four key m^6^A regulatory genes. The R2: Genomics Analysis and Visualization Platform (https://hgserver1.amc.nl/cgi‐bin/r2/main.cgi) was employed to analyze the survival of the most significant modular genes. Kaplan–Meier plotter (http://kmplot.com/) was employed to analyze recurrence‐free survival (RFS) of gene. SRAMP (http://www.cuilab.cn/sramp/)^26^ was used to determine the m^6^A modification site of the target gene. The RMbase database (http://rna.sysu.edu.cn/rmbase/) was employed to identify the gene m^6^A motifs and modification peak.

### 2.5 Clinical samples

Twenty paraffin‐embedded pancreatic cancer specimens and paired adjacent normal pancreatic tissue specimens were obtained from Renmin Hospital of Wuhan University from September 1, 2019, to August 31, 2020, and clinicopathological characteristics of the clinical samples were shown in Table S1. The main inclusion criterion was histopathologically confirmed pancreatic cancer. The main exclusion criteria were other malignant tumors and neoadjuvant therapy. This study obtained approval from the Ethics Committee of Renmin Hospital of Wuhan University. The tissue samples used in this study have received written informed consent from the patients.

### 2.6 Cell culture

PC cell lines Panc‐1 and SW1990 were all taken from National Collection of Authenticated Cell Cultures (Shanghai, China). All cells were cultured in DMEM (Hyclon) supplemented with 10% FBS and 1% penicillin/streptomycin (Invitrogen; Thermo Fisher Scientific, Inc.) at 37°C with 5% CO_2._

### 2.7 siRNA transfection

PC cells (2 × 10^5^ cells/well) were planted in a six‐well plate 24 h before transfection, so the cell density can reach 40~50% confluence during transfection. Then the cells were transfected with small interfering RNA [siRNA; for human FTO (5'‐GUGGCAGUACAGUUAUATT‐3') or equivalent negative control siRNA (5'‐UUCUCCGAACGUGUCACGUTT‐3')] (GenePharma, Shanghai, China) using a high‐efficiency transfection reagent (SignaGen, Maryland, USA). The transfection concentration of si‐FTO and si‐NC is 100 nmol/L. After 48 h of transfection, cells were collected for subsequent assays.

### 2.8 Immunohistochemistry (IHC) analysis

IHC was performed as previous described^27^ and was employed to determine the expression of FTO protein in PC clinical specimens. The tissues were fixed with 4% paraformaldehyde, embedded in paraffin, and sectioned at 3 μm. Then use rabbit polyclonal anti‐THBS2 antibody (1:250, abclonal, A8561, China), ADAMTS2 Antibody (1:25, Invitrogen, PA5‐50539, USA), COL12A1 (1:50, abcam, ab121304, UK), and anti‐FTO antibody (1:250, abclonal, A3861, China) at 4°C overnight. After the secondary antibody was incubated, the sections were stained with the DAB reagent. The results were analyzed using Image‐Pro Plus 6.0 software.

### 2.9 Western blotting

RIPA buffer (Beyotime, China) was used to isolate total protein from cells. SDS‐PAGE was used to isolate the same amount of protein and transfer it to the PVDF membrane. Block the membrane with 5% skim milk at 20°C for 1 h, then use primary antibodies: FTO (1:1000, abclonal, A3861, China), COL12A1 (1:1000, abcam, ab121304, UK), THBS2 (1:1000, abclonal, A8561, China), ADAMTS2 (1:1000, abclonal, A10272, China), ALKBH5 (1:2000, proteintech, 16837‐1‐AP, China), GAPDH (1:1000, biosharp, BL006A, China). Then wash the membrane with TBST buffer and mix it with horseradish peroxidase‐labeled goat anti‐rabbit secondary antibody (1:5000, biosharp, BL003A, China) or goat anti‐mouse secondary antibody (1:5000, biosharp, BL001A, China) at 20°C for 60 min. Membrane analysis using ECL kit (biosharp, China). The results were analyzed with ImageJ (version 1.50 g) software.

### 2.10 Wound healing experiment

PC cells (2 × 10^6^ cells/well) were seeded in 6‐well plates until they reached ~100% confluence. The cell monolayer was scratched with a 100 μl sterile pipette tip and then rinsed with PBS. Subsequently, the cells were incubated in serum‐free DMEM, and images were captured using an inverted optical microscope (magnification ×100) at the time points of 0 and 48 h.

### 2.11 Migration and invasion assays

Transwell chambers (Corning, USA) with 8‐μm‐pore size membranes were utilized to determine the invasiveness and migration ability of PC cells. For invasion assay, Matrigel (BD Biosciences, USA) was applied to the upper chamber of Transwell in advance at 37°C for 4 h. After siRNA transfection, si‐FTO, or corresponding si‐NC PC cells (5 × 10^4^ cells/well) were planted in the upper chamber with serum‐free DMEM medium in a 24‐well plate, and 20% FBS medium was added in the lower chamber. The cells were cultured for 24 h (migration assay) and 48 h (invision assay) at 37°C. Transmembrane cell count of 5 random fields was performed using an inverted optical microscope (magnification, ×100; Olympus).

### 2.12 Quantitative real‐time polymerase chain reaction (qRT‐PCR)

Total RNA was isolated from cells using Trizol (Thermo Fisher Scientific, USA). qRT‐PCR was conducted as described in a previous study.^28^ ADAMTS2, COL12A1, and THBS2 were normalized against GAPDH. The RNA expression level was analyzed using 2^−ΔΔCT^. The primer sequences utilized in this study were presented in supplemental Table S2.

### 2.13 Dot blot assay

RNA m^6^A Dot blot was conducted as described formerly,^29^ to detect the level of total mRNA m^6^A modification after knockdown of FTO. Briefly, poly (A) RNA (400 ng) was dotted on a nitocellulose membrane (Millipore, USA). The membrane was then UV cross‐linked (254 nm), blocked, and incubated with m^6^A antibody (1:250, Abcam, ab208577, UK) at 4°C overnight. After being incubated with the secondary antibody at 20°C for 60 min, the membrane was analyzed using enhanced chemiluminescence (ECL) kit (BioSharp, China).

### 2.14 Methylated RNA immunoprecipitation (MeRIP) assay

Cells were collected and treated with polyester lysis buffer [150 mM KCl, 20 mM MgCl2, 10 mM HEPES (pH 7.0), 0.5% NP40, 0.5 mM DTT, 100 U/ml RNase inhibitor and protease inhibitor cocktail] in the lysis for 10 min. Draw 1/10 suspension as input. Then add the lysate to an EP tube containing 5 μg of m^6^A antibody (Abcam, UK) or IgG control‐magnetic beads (MCE, China) and incubate overnight at 4°C. After the incubation period, wash with binding/washing buffer (PBS pH 7.4, 1% Triton X‐100) three times, 5 min each time. The immunoprecipitated RNA was extracted using TRIzol reagent (Invitrogen, USA). And perform qRT‐PCR detection on RNA as described above.

### 2.15 Statistics analysis

The data were expressed as mean ± SD. All experiments were performed at least thrice. The experimental data were analyzed using GraphPad Prism v8.4.0, SPSS v25.0 (IBM) and R software. Unpaired Student's *t*‐test or one‐way analysis of variance was utilized for statistical differences. The difference is statistically significant with *p* < 0.05, and the results are as follows: *p < 0.05, **p < 0.01, ***p < 0.001, no statistical significance (NS).

## 3 RESULTS

### 3.1 Identification of m^6^A‐related genes in PC

**FIGURE 2:**
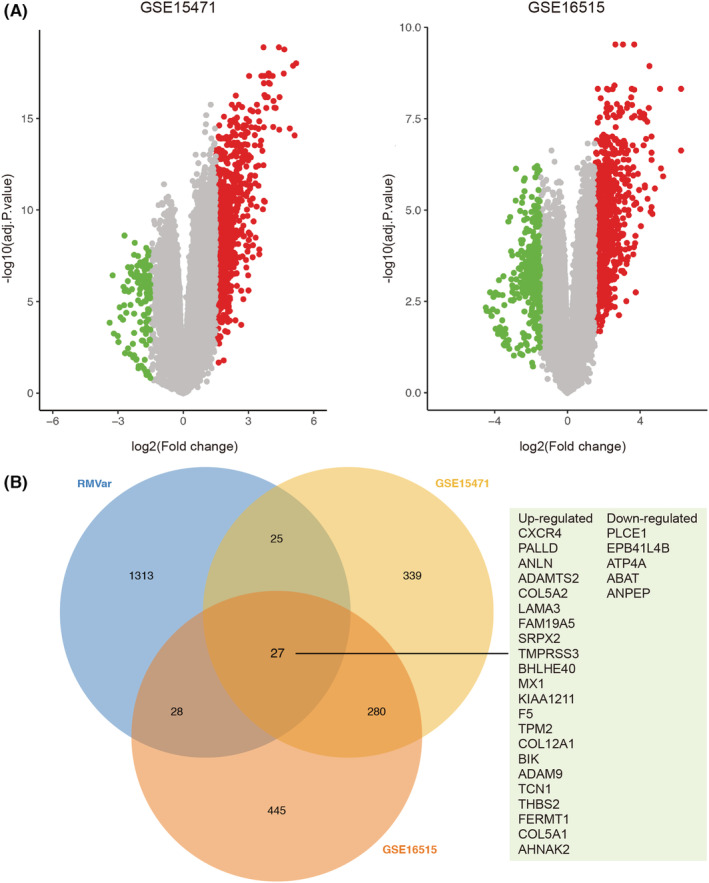
Identification of m^6^A‐related genes in pancreatic cancer. (A) The volcano map constructed by imageGP online server (http://www.ehbio.com/ImageGP/) showed that 671 and 780 differentially expressed genes (DEGs) were identified in the GSE15471 and GSE16515 datasets, respectively. (B) Venn diagram showed that 27 common genes were obtained by overlapping the DEGs with 1393 m^6^A‐related genes associated with pancreatic adenocarcinoma (PAAD) were obtained from RMVar database.

We obtained the gene expression sets of GSE15471 and GSE16515 from the GEO database. The volcano map constructed by imageGP online server (http://www.ehbio.com/ImageGP/) showed that 671 and 780 DEGs were identified in the GSE15471 and GSE16515 datasets, respectively (Figure 2A). A total of 27 common genes were obtained by overlapping the DEGs with 1393 m^6^A‐related genes associated with PAAD were obtained from RMVar database (Figure 2B).

### 3.2 PPI network construction and module analysis

**FIGURE 3:**
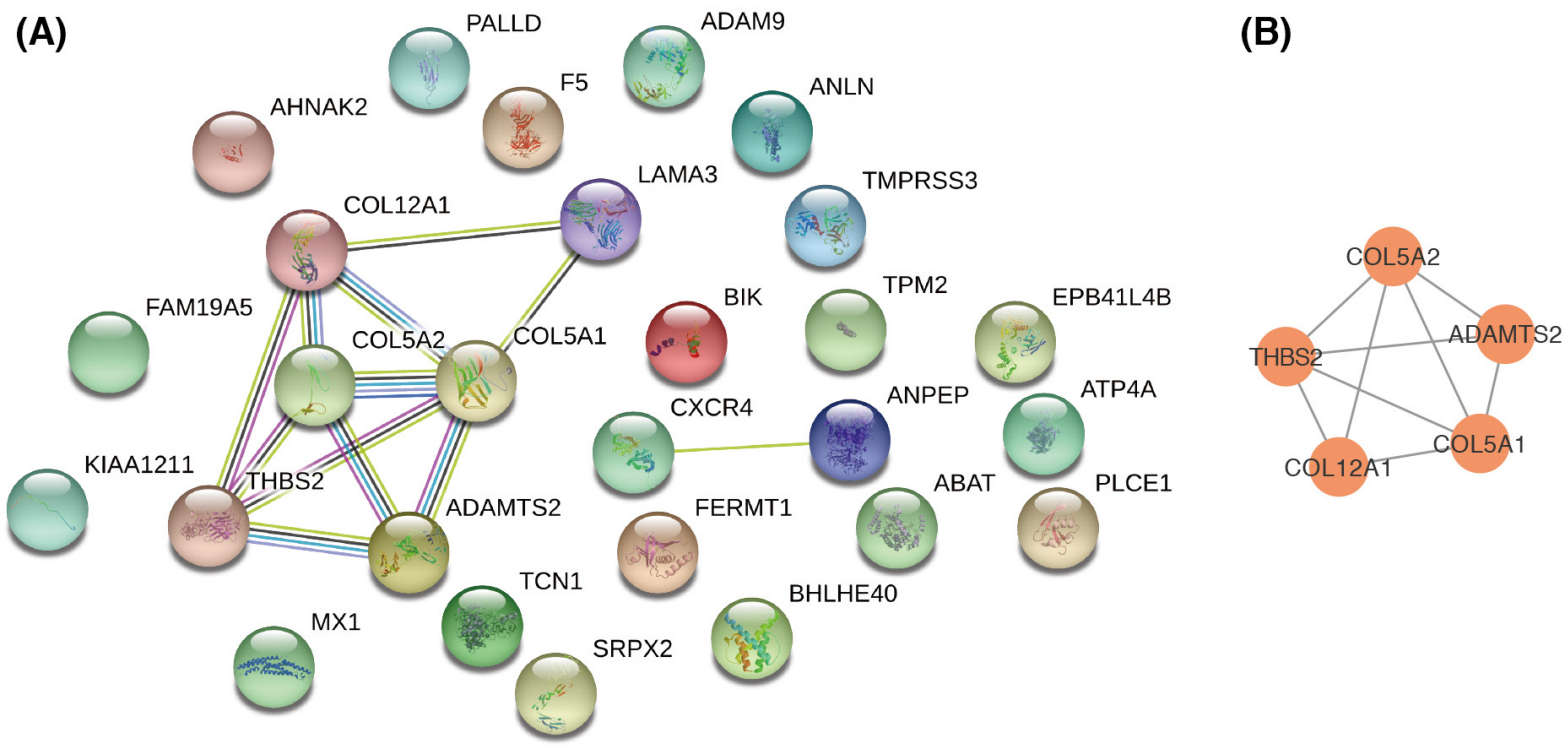
PPI network construction and module analysis. (A) PPI network of 27 m6A‐related genes was built using the STRING database. (B) The most significant module was obtained through Cytoscape MCODE plug‐in analysis.

To better understand the relationship between 27 m^6^A‐related genes, we built a PPI network using the STRING database (Figure 3A). The most significant module was obtained through Cytoscape MCODE plug‐in analysis (Figure 3B). The most important modules include five genes, ADAMTS2, COL5A1, COL5A2, COL12A1, and THBS2. GEPIA analysis further confirmed that these hub genes expression were remarkably increased in PC tissues compared with normal pancreatic tissues (Figure S1).

### 3.3 GO and KEGG analysis of hub genes

**FIGURE 4:**
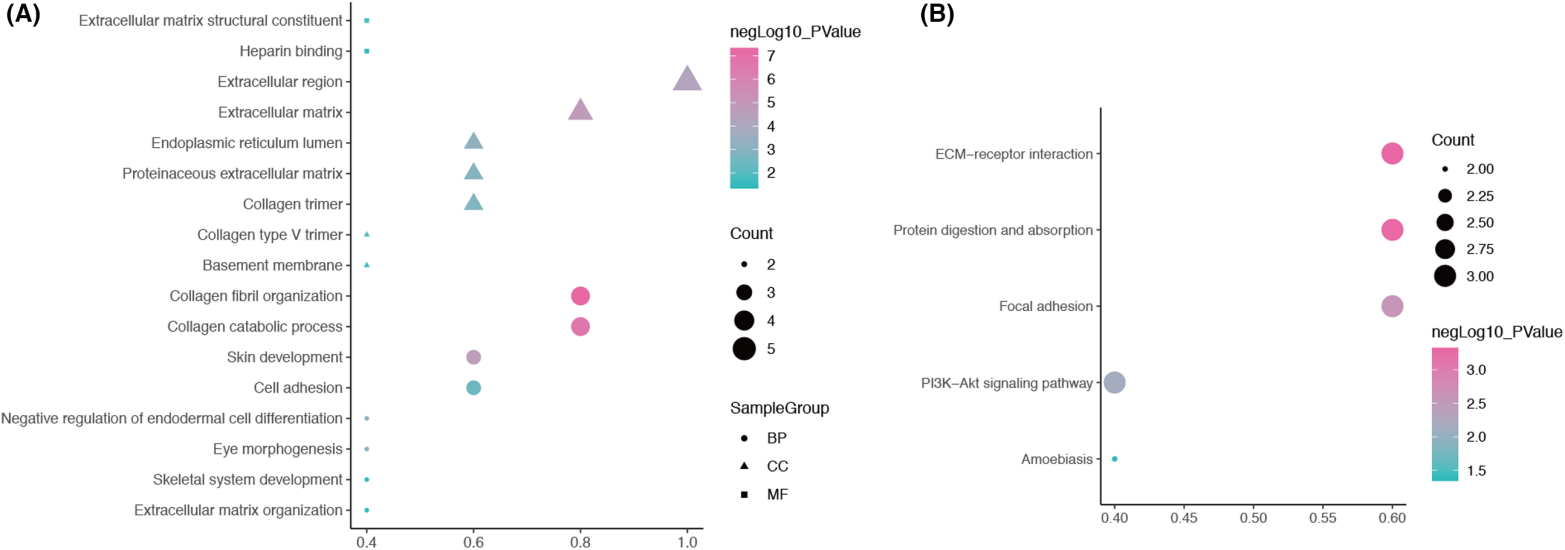
GO and KEGG analysis of hub genes. (A) GO enrichment analysis showed biological process (BP), cell composition (CC), and molecular function (MF) of hub genes. (B) KEGG pathway analysis indicated that the target was obviously concentrated in ECM‐receptor interaction, protein digestion, and absorption, focal adhesion and PI3K‐Akt signaling pathway.

To better study the biological classification of the five hub genes, DAVID was used to conduct the function and pathway enrichment analysis. The results (Figure 4A) revealed that the biological process (BP) of hub genes were mainly concentrated in collagen fibril organization, collagen catabolic process, skin development, cell adhesion, negative regulation of endodermal cell differentiation, eye morphogenesis, skeletal system development, ECM organization. Changes in cell composition (CC) were mainly concentrated in extracellular region, ECM, proteinaceous ECM, collagen trimer, collagen type V trimer, and basement membrane. The changes in molecular function (MF) were mainly concentrated in ECM structural constituent and heparin binding. As shown in Figure 4B, the KEGG pathway analysis suggested that the hub genes were obviously concentrated in ECM‐receptor interaction, protein digestion, and absorption, focal adhesion and PI3K‐Akt signaling pathway.

### 3.4 Key m^6^A‐related genes and m^6^A regulator selection and analysis

**FIGURE 5:**
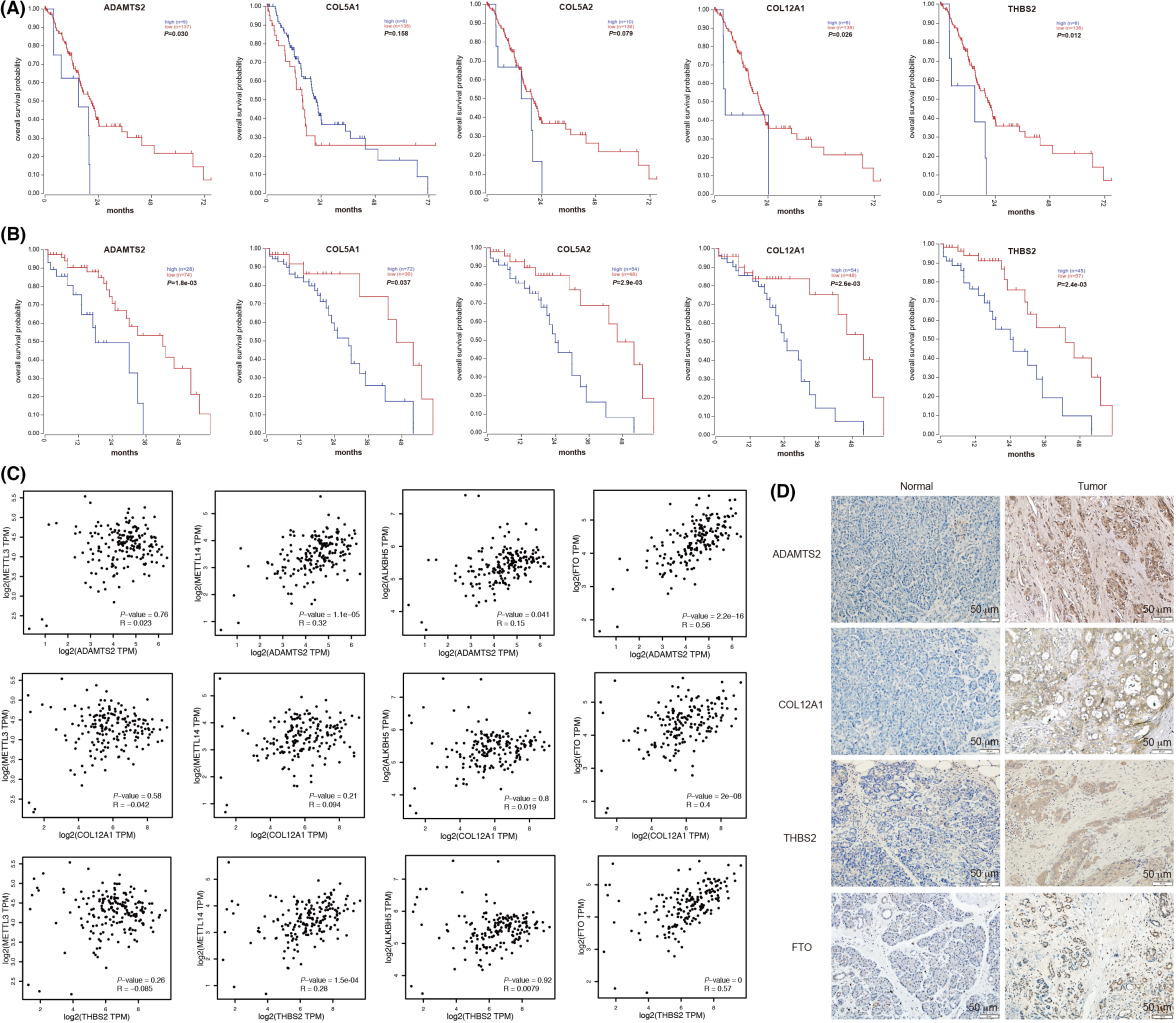
Key m^6^A‐related genes and m^6^A regulator selection and analysis. (A and B) Survival analysis showed that the high expression of ADAMTS2, COL12A1, and THBS2 were related to poor prognosis both in TCGA (A) and Yeh’ data (B). (C) GEPIA correlation analysis showed the correlation between m^6^A‐related genes and the main m^6^A regulators including m^6^A RNA methyltransferase METTL3 and METLL14 and m^6^A RNA demethylase FTO and ALKBH5. (D) The IHC analysis indicated that ADAMTS2, COL12A1, THBS2, and FTO were overexpressed in pancreatic cancer compared with normal pancreas (magnification, ×200).

Survival analysis showed that the over‐expression of ADAMTS2, COL12A1, and THBS2 was related to poor prognosis both in TCGA (PAAD) (Figure 5A) and Yeh' data^30^ (Figure 5B), which aroused our interest. Moreover, the Kaplan–Meier plotter analysis revealed that high expression of ADAMTS2, COL12A1, and THBS2 were related to RFS of PAAD (Figure S2). GEPIA correlation analysis was used to analyze the correlation between m^6^A‐related genes and the main regulators of m^6^A including m^6^A RNA methyltransferase (METTL3, METLL14) and m^6^A RNA demethylase (FTO, ALKBH5) in the TCGA database. The data revealed that ADAMTS2, COL12A1, and THBS2 were positively correlated with FTO (Figure 5C). The results indicated that these three genes may play important roles in m^6^A modification of PC. The IHC analysis confirmed that ADAMTS2, COL12A1, and THBS2 were overexpressed in PC (Figure 5D and Figure S3A–C). Moreover, IHC analysis suggested that FTO was overexpressed in PC (Figure 5D and Figure S3D), consisted of GEPIA analysis (Figure S4) and previous studies. FTO has been reported to facilitate PC cell proliferation by m^6^A modification.^31^ Herein, we hypothesized that FTO may promote the progression of PC by regulating ADAMTS2, COL12A1, and THBS2 expression in an m^6^A‐dependent manner.

### 3.5 FTO facilitates migration and invasion ability of PC cells

**FIGURE 6:**
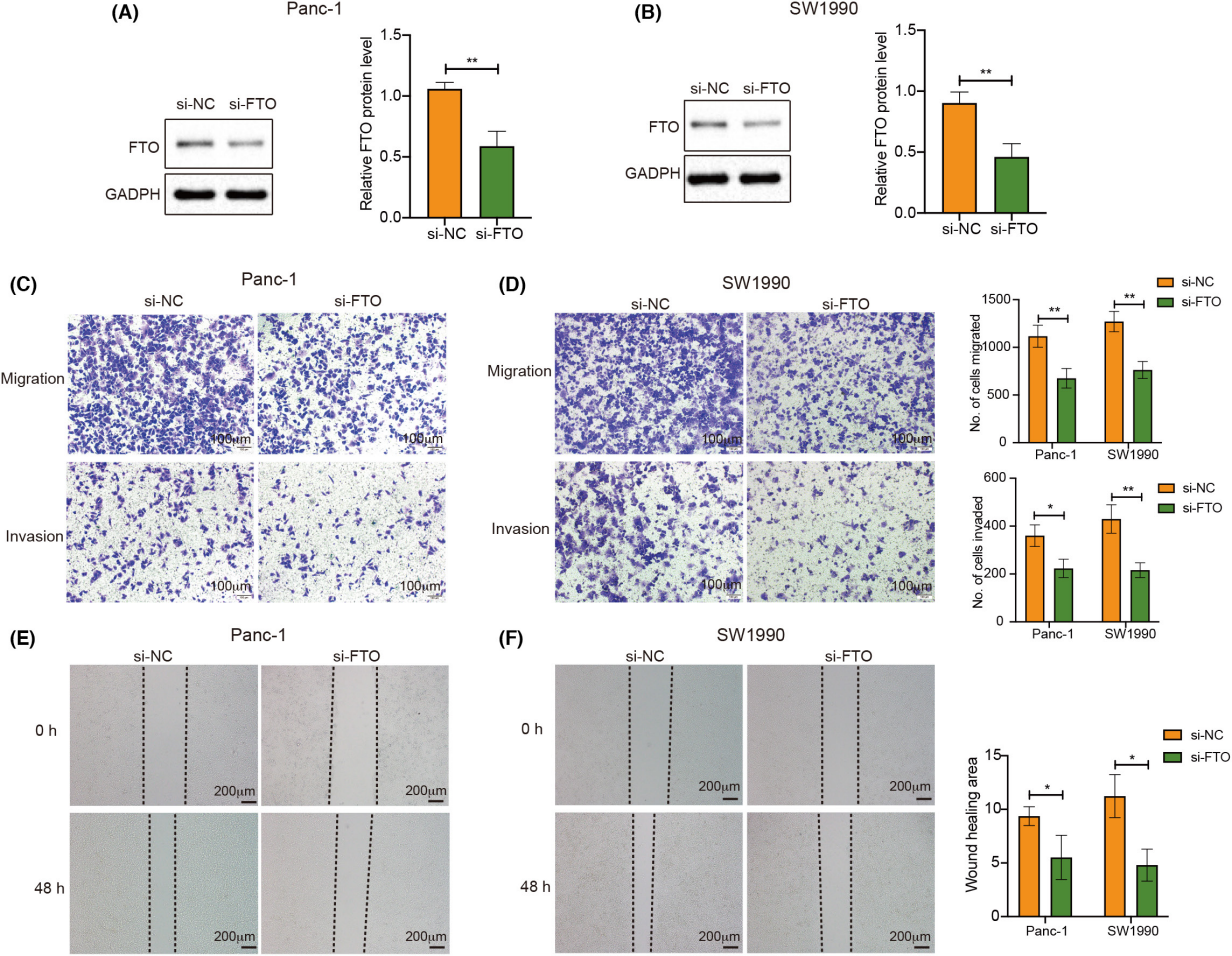
FTO promotes migration and invasion ability of pancreatic cancer cells. (A and B) Western blotting analysis revealed significant knockdown efficiency of si‐FTO in Panc‐1 and SW1990 cell lines. (C and D) Migration and invasion assays revealed that knockdown of FTO suppressed the migration and invasion of pancreatic cancer cells (magnification, x100) (Figure 6C and D). (E and F) Wound healing experiment revealed that inhibition of FTO restrained the migration of pancreatic cancer cells (magnification, x40) (Figure 6E and F). *p < 0.05, **p < 0.01.

In order to investigate the function of FTO in PC, we used small interfering RNA to knockdown FTO in Panc‐1 and SW1990 cell lines. Western blotting analysis revealed significant knockdown efficiency (Figure 6A and B). KEGG analysis indicated that ADAMTS2, COL12A1, and THBS2 are closely related to ECM, and ECM plays an important role in tumor metastasis and progression.^32^, ^33^ Therefore, we paid attention to the effect of FTO on the metastasis of PC cells. Migration experiments showed that knockdown of FTO suppressed the migration of PC cells (Figure 6C and D). The wound healing experiment further validated that inhibition of FTO restrained the migration of PC cells (Figure 6E and F). Invasion assay revealed that downregulation of FTO inhibited the invasion capacity of PC cells (Figure 6C and D). Taken together, the data suggested that FTO facilitates PC cells abilities to migrate and invade.

### 3.6 ADAMTS2, COL12A1, and THBS2 may be the direct target genes of m^6^A RNA demethylase FTO

**FIGURE 7:**
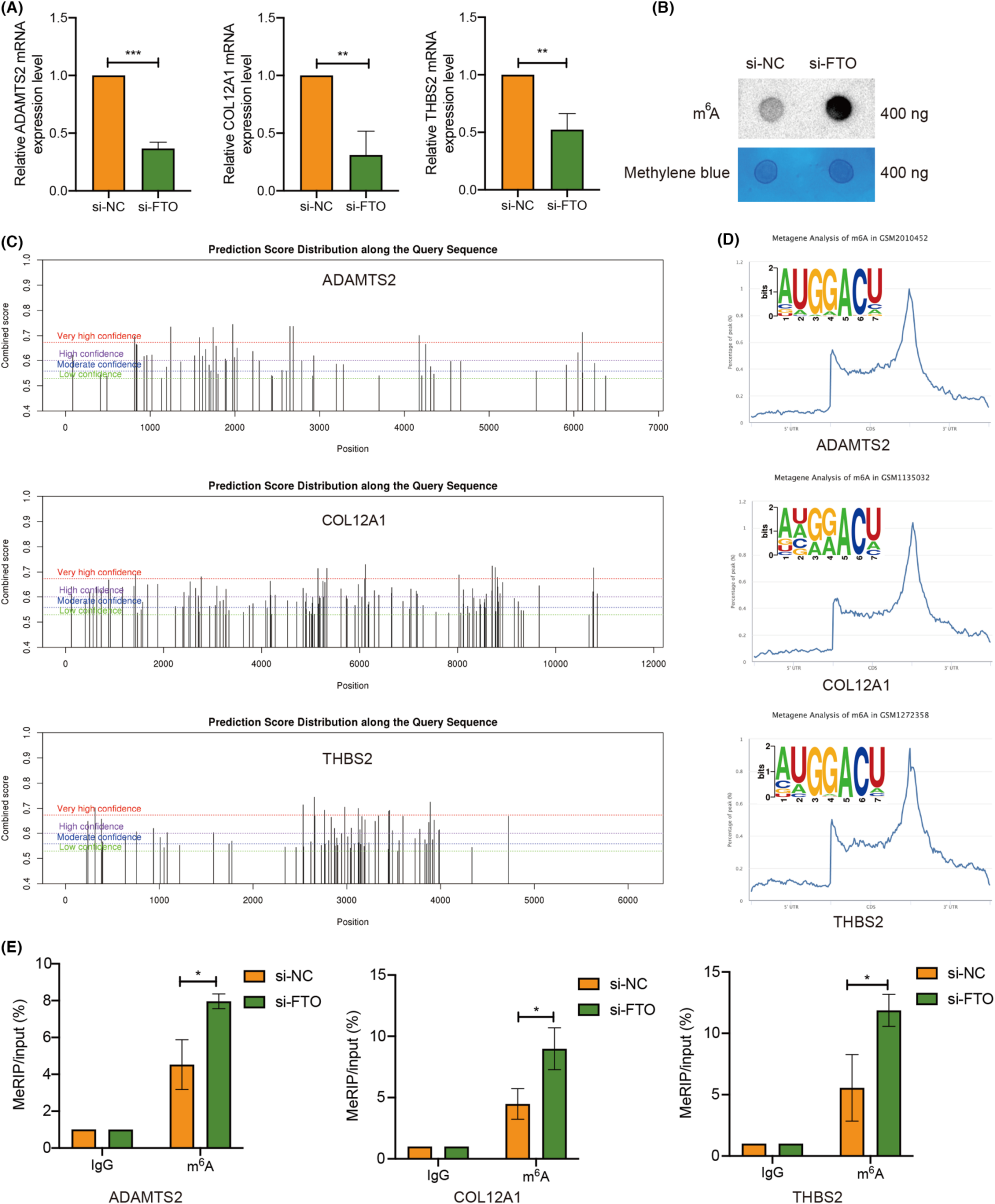
ADAMTS2, COL12A1 and THBS2 are the direct target genes of m^6^A RNA demethylase FTO. (A) qRT‐PCR showed that the mRNA levels of ADAMTS2, COL12A1, and THBS2 in siFTO Panc‐1 cell line were significantly down‐regulated compared with the si‐NC group. (B) Dot blot showed that m^6^A level of pancreatic cancer cells was increased after knocking down FTO. (C) The SRAMP database predicted that m^6^A modification sites of ADAMTS2, COL12A1, and THBS2. (D) Motifs and metagene analyses from RMbase database revealed the m^6^A motifs enrichment and modification peak of ADAMTS2, COL12A1, and THBS2. (E) MeRIP analysis indicated that FTO knockdown remarkably increased the m^6^A level of ADAMTS2, COL12A1, and THBS2 mRNA. *p < 0.05, **p < 0.01, ***p < 0.001.

In order to verify the relationship between FTO and key genes, qRT‐PCR was employed to determine the changes in mRNA levels of key genes after knocking down FTO in Panc‐1 cell line. The results indicated that compared with the NC group, the mRNA and protein levels of the key genes were significantly down‐regulated (Figure 7A and Figure S5). Meanwhile, FTO knockdown did not affect ALKBH5 protein expression (Figure S6). Dot blot showed that m^6^A level of PC cells was increased after knocking down FTO (Figure 7B). The SRAMP database predicted that m^6^A modification sites of ADAMTS2, COL12A1, and THBS2 (Figure 7C), and motifs and metagene analysis from RMbase database revealed the m^6^A motifs enrichment and modification peak of ADAMTS2, COL12A1, and THBS2 (Figure 7D). These data suggest that ADAMTS2, COL12A1, and THBS2 may be downstream targets of FTO in PC. Moreover, MeRIP analysis showed that FTO knockdown remarkably increased the m^6^A level of ADAMTS2, COL12A1, and THBS2 mRNA, indicating that ADAMTS2, COL12A1, and THBS2 may be the direct target genes of m^6^A RNA demethylase FTO.

## 4 DISCUSSION

Recently, several studies have reported m^6^A modulated ECM degradation and involved the progression of osteoarthritis.^34^, ^35^ Here, we identified for the first time the regulatory role of m^6^A modification in the regulation of ECM of PC. Studies have confirmed that ECM components of solid tumors are associated with metastasis and progression.^32^, ^36^ A large number of ECMs are the hallmark of pancreatic ductal adenocarcinoma (PDAC),^37^ and ECM has basic oncogenic characteristics and is a major factor in promoting the progress of PDAC and limiting anti‐tumor therapy.^38^ However, ECM exists as a complex macromolecular substrate, and it is not clear how its molecular composition contributes to cancer progression. The components of ECM continuously interact with epithelial cells through ligands that act as cell receptors to transmit signals that modulate cell biological behavior such as adhesion, migration, proliferation, apoptosis, or differentiation.^39^ In addition, cells continuously rebuild and reshape ECM through synthesis, degradation, recombination, and chemical modification.^40^ Emerging evidence suggests that changes in the activity of ECM regulators in cancer can trigger pathological ECM remodeling, promote metastatic transmission, and make ECM regulators a potential target for cancer treatment.^32^ In the present study, GO analysis found that cell component of hub genes was mainly enriched in ECM, and molecular function was mainly concentrated in ECM structural constituent. KEGG analysis revealed that hub genes were concentrated in ECM‐receptor interaction, indicating that hub genes were closely correlated with ECM. Moreover, survival analysis indicated that key genes were related to poor prognosis of PC. Further bioinformatics analysis and validation experiments revealed that key genes ADAMTS2, COL12A1, and THBS2 mRNA had significant m^6^A modification sites and were regulated by m^6^A RNA demethylase FTO. These results indicate that m^6^A methylation, an emerging molecular mechanism for post‐transcriptional regulation, is involved in the regulation of tumor ECM.

In the present study, we identified three important m^6^A modification‐related target genes ADAMTS2, COL12A1, and THBS2 associated with OS and RFS of PC by using the integrated bioinformatics analysis. GEPIA correlation analysis indicated that only FTO was positively correlated with ADAMTS2, COL12A1, and THBS2 among the main m^6^A regulatory factors. FTO plays a carcinogenic role in a variety of cancers, providing an opportunity to develop effective targeted therapeutics.^41^ Tao et al.^42^ identified that FTO acts as a biomarker for the diagnosis or prognosis of bladder cancer and promotes the tumorigenesis of bladder cancer via regulating the m^6^A level of MALAT. Xu et al.^43^ revealed that FTO promotes breast cancer cell invasion and migration through miR‐181b‐3p/ARL5B axis. In another study, Tang et al.^31^ found that using FTO small interfering RNA (siFTO) to treat PC cells can inhibit cell proliferation and promote cell apoptosis. Interestingly, Zeng et al.^44^ declared that FTO suppresses PC tumorigenesis by demethylating PJA2 and inhibiting Wnt signaling. It is worth noting that SW1990 cells were cultured without CO_2_ in this study, cell culture under different conditions may lead to differences in experimental results. Similarly, Ruan et al.^45^ found that FTO downregulation mediated by hypoxia aggravates colorectal cancer metastasis, it indicates that FTO plays a cancer suppressor role in colorectal cancer. However, a recent study reported that FTO facilitates colorectal cancer progression and chemotherapy resistance by demethylating G6PD/PARP1.^46^ These studies indicate that FTO may play a complex dual role in tumorigenesis. In the current study, we found that knocking down FTO‐inhibited PC cell migration and invasion. In addition, knockdown FTO decreased the mRNA expression levels of ADAMTS2, COL12A1, and THBS2. MeRIP experiment further confirmed that ADAMTS2, COL12A1, and THBS2 mRNA were the target mRNAs of FTO in an m^6^A modification‐dependent manner. These data indicate that the highly expressed FTO in PC promotes cell migration and invasion at least through upregulating the expression of ADAMTS2/COL12A1/THBS2 via m^6^A modification.

ADAMTS2, a member of the ADAMTS family, is a procollagen N‐protease. ADAMTS regulates its activity and regulates its substrate binding preference depending on its carboxy‐terminal helper region's association with ECM. Studies have shown that ADAMTS2 expression is abnormally increased in a variety of cancers. Jiang et al.^47^ found that ADAMTS2 overexpression in gastric cancer cells and stroma predicted poor clinical prognosis. Tota et al.^48^ reported abnormal up‐regulation of ADAMTS2 gene expression in T/myeloid hybrid acute leukemia. A recent study revealed that miR‐29A was observed to inhibit the transformation of several key pro‐tumorigenesis and fibrosis targets, including ADAMTS2, in pancreatic stellate cells in PC.^49^ This study further examined the regulatory role of miR‐29A in PDAC ECM remodeling and tumor‐matrix crosstalk. In the present study, we found that ADAMTS2 was regulated by m^6^A demethylase FTO and may be involved in PC metastasis.

COL12A1 encodes the alpha chain of type XII collagen, and collagen XII is an important ECM protein, which forms a microfilament mesh and interacts with other ECM molecules to provide structural support for cells.^50^ Multiple studies have reported that collagen XII triggers signaling pathways to modulate cell migration and invasion^51^ and tumor growth.^52^ It was found that COL12A1 expression was abnormally elevated in ovarian cancer (OC), and overexpression of COL12A1 could also induce drug resistance in OC cell lines.^53^, ^54^ In addition, Verghese et al.^55^ reported that COL12A1 was overexpressed in breast cancer matrix and significantly correlated with tumor recurrence, and down‐regulated miR‐26B targeting COL12A1 in breast cancer enhanced cell abilities to migrate and invade. This study indicates that COL12A1 can be post‐transcriptionally regulated by microRNA. Here, we revealed that a novel post‐transcriptional regulatory mechanism, m^6^A modification, is involved in COL12A1 regulation.

THBS2 is a glycoprotein that may act as an angiogenic inhibitor, mediating cell–cell and cell‐matrix interactions. Mutations in the THBS2 gene in mice increased susceptibility to cancer.^56^ Recent studies have found that THBS2 promotes tumor progression. For example, circular RNA circ_0020123 regulates THBS2 through sponge miR‐590‐5p to facilitate non‐small cell lung cancer cell proliferation and migration and suppress cell apoptosis.^57^ Another study showed THBS2 promotes proliferation and metastasis of colon cancer cell line HCT116.^58^ Recent studies have reported that the protein THBS2 can accurately diagnose PDAC.^59^, ^60^ Furthermore, THBS2 is more strongly expressed in advanced PC than pancreatic intraepithelial neoplasia, and the THBS2/CA19‐9 marker panel may be helpful in detecting resectable early tumors.^60^ These findings suggest that THBS2 may play a carcinogenic role in PC. In the current study, we found that THBS2 may be associated with PC cell migration and invasion modulated by FTO. Interestingly, Zou et al.^61^ found that low THBS2 expression in ovarian cancer patients may be more likely to lead to tumor metastasis. This indicates that THBS2 may play an interesting dual role in different types of tumors.

We acknowledge there may be some limitations of this study. Firstly, in general, FTO acts as an m^6^A demethylase to reduce the m^6^A modification level of target mRNA and then affects the expression of target gene through specific reader proteins. The present study lacks the investigation of regulatory role of m^6^A readers in key genes in terms of mechanism, which is of great significance to the integrity of the study and need to be further explored in the future. Secondly, this study investigated the correlation between ADAMTS2, COL12A1, and THBS2 and the prognosis of PC, but lacked in‐depth investigation on their specific roles in the malignant phenotype of PC cells. In addition, only two PC cell lines were selected for in vitro experiments in this study, and more PC cell lines are needed for broader validation of the results.

In conclusion, we identified the important role of m^6^A modification in the regulation of ECM‐related molecules in PC for the first time. Our data revealed that ADAMTS2, COL12A1, and THBS2 may be involved in PC metastasis and progression, and this role is regulated by the m^6^A demethylation transferase FTO. These molecules hold promise as novel biomarkers of clinical prognosis in PC and may be served as potential therapeutic targets of PC. The present study provides a new perspective for the development of therapeutic strategies for PC.

## AUTHOR CONTRIBUTIONs

Conceptualization, Wei Wang and Zhi‐Gang Tang; Funding acquisition, Zhi‐Gang Tang; Methodology, Ying He; Project administration, Kai‐Huan Yu and Zhi‐Gang Tang; Software, Wei Wang; Supervision, Kai‐Huan Yu and Zhi‐Gang Tang; Validation, Wei Wang and Ying He; Visualization, Wei Wang; Writing ‐ original draft, Wei Wang and Ying He; Writing ‐ review & editing, Lun Wu, Lu‐Lu Zhai, Long‐Jiang Chen, and Li‐Chao Yao.

## FUNDING INFORMATION

This study was supported by the Fundamental Research Funds for the Central Universities (2042022kf1100), Foundation of Health Commission of Hubei Province (grant no. WJ2021M063 and WJ2021F052), and National Natural Science Foundation of China (81272740).

## CONFLICT OF INTEREST

There are no potential conflict of interest to declare.

## ETHICS STATEMENT

This study was conducted in accordance with the Declaration of Helsinki (as revised in 2013). The study obtained approval from the Ethics Committee of Renmin Hospital of Wuhan University (No. WDRY2019‐K070).

## Supporting information


Figure S1

Figure S2

Figure S3

Figure S4

Figure S5

Figure S6

Table S1

Table S2

## Data Availability

The data presented in this study are available upon request from the corresponding author.
