# Supplementary material for: N6 ‐methyladenosine RNA demethylase FTO regulates extracellular matrix‐related genes and promotes pancreatic cancer cell migration and invasion
Source: Cancer Med. 2022 Jul 25;12(3):3731–43. doi: 10.1002/cam4.5054 (PMC9939218; doi:10.1002/cam4.5054)
Supplement: Supplementary file 7 — Table S1 [file CAM4-12-3731-s006.docx]

**Table S1. Clinicopathological characteristics of the clinical samples**

| Clinicopathological characteristics | Patients  (n=20) |
| --- | --- |
|  |  |
| Age (years) |  |
| ≤50 | 7 |
| >50 | 13 |
| Gender |  |
| male | 12 |
| female | 8 |
| Histological grading |  |
| Low differentiation | 13 |
| High differentiation | 7 |
| Tumor diameter |  |
| ≤2cm | 6 |
| >2cm | 14 |
| TNM staging |  |
| I | 3 |
| II | 9 |
| III | 8 |
| Lymph node |  |
| Positive | 13 |
| Negative | 7 |
| PNI |  |
| Positive | 15 |
| Negative | 5 |
