# Supplementary material for: N6 ‐methyladenosine RNA demethylase FTO regulates extracellular matrix‐related genes and promotes pancreatic cancer cell migration and invasion
Source: Cancer Med. 2022 Jul 25;12(3):3731–43. doi: 10.1002/cam4.5054 (PMC9939218; doi:10.1002/cam4.5054)
Supplement: Supplementary file 8 — Table S2 [file CAM4-12-3731-s007.docx]

**Table S2. Primer sequences for RT-qPCR**

| Genes | Sequences |
| --- | --- |
| ADAMTS2 | Forward: 5’-TGCACCTGGCAAGCATTGTT-3’ |
|  | Reverse: 5’-GAGCCAAACGGACTCCAAGC-3’ |
| COL12A1 | Forward: 5’-AGTGTGCCAGCATCCCATAC-3’ |
|  | Reverse: 5’-CACGTGCGCAAACATCTCAG-3’ |
| THBS2 | Forward: 5’-GGAAGAGCCAGACAGAGTGA-3’ |
|  | Reverse: 5’-CTTTGCATAGCCCGACCTTC-3’ |
| GAPDH | Forward: 5’-CATCATCCCTGCCTCTACTGG-3’ |
|  | Reverse: 5’-GTGGGTGTCGCTGTTGAAGTC-3’ |
